# Feasibility and Concordance of a Large Language Model (ChatGPT-5) as a Clinical Decision Support Tool in Gynecologic Oncology Tumor Boards: A Blinded, Multi-Observer Study

**DOI:** 10.3390/jcm15124451

**Published:** 2026-06-09

**Authors:** Hatice Asoglu, Sevgul Kose, Oguzhan Kayim, Ali Abaci, Ghanim Khatib, Mehmet Ali Vardar, Emine Kilic Bagir, Derya Gumurdulu, Mehmet Mutlu Kidi, Ertugrul Bayram, Tolga Koseci, Berksoy Sahin, Ismail Oguz Kara

**Affiliations:** 1 Department of Medical Oncology, Cukurova University Faculty of Medicine, 01330 Adana, Turkey; 2 Department of Radiology, Cukurova University Faculty of Medicine, 01330 Adana, Turkey; 3 Department of Gynecologic Oncology, Cukurova University Faculty of Medicine, 01330 Adana, Turkey; 4 Department of Pathology, Cukurova University Faculty of Medicine, 01330 Adana, Turkey

**Keywords:** ChatGPT-5, large language model, gynecologic oncology, tumor board, artificial intelligence, clinical decision support, reproducibility

## Abstract

**Background:** The reliability of large language models (LLMs) in complex oncologic decision-making remains inadequately validated. This study evaluated the concordance of ChatGPT-5 with multidisciplinary tumor board (MDT) decisions in gynecologic oncology, assessing accuracy, reproducibility, and domains of discordance. **Methods:** We analyzed 242 gynecologic cancer cases (endometrial *n* = 102, ovarian *n* = 85, cervical *n* = 40, rare *n* = 15) discussed at the Çukurova University Gynecologic Oncology MDT (2024–2025). Standardized clinical summaries were input into ChatGPT-5 using a structured prompt template. Each case was queried three times within a single calendar day using independent conversations. Recommendations were evaluated by two blinded medical oncologists using a 5-point Likert scale. A composite performance score (CPS) was calculated as (mean Likert/5) × 100. Concordance was analyzed using Cohen’s kappa (κ). **Results:** Inter-rater reliability was substantial to almost perfect for both MDT (κ = 0.761) and AI (κ = 0.814) evaluations (both *p* < 0.001). MDT–AI concordance was fair (Rater 1: κ = 0.258; Rater 2: κ = 0.334). CPS were significantly higher for MDT versus AI (Rater 1: 93.8% ± 5.2 vs. 89.4% ± 6.7; Rater 2: 93.4% ± 5.5 vs. 89.7% ± 6.4; both *p* < 0.001). Full consistency across three queries was achieved in only 37.2% of cases (90/242). AI performance was significantly inferior in advanced-stage disease (*p* = 0.008), genetic testing (*p* = 0.006), fertility-sparing (*p* = 0.018), and novel therapeutics (*p* = 0.003). **Conclusions:** ChatGPT-5 demonstrates potential as a clinical decision support tool but lacks sufficient reliability for independent use. Key limitations include inconsistency in 62.8% of cases, suboptimal performance in advanced-stage disease, and deficiencies in precision oncology domains. These findings suggest that human expertise remains indispensable for the individualized management of complex gynecologic malignancies.

## 1. Introduction

Artificial intelligence (AI) has emerged as a transformative force in healthcare, with large language models (LLMs) representing one of the most significant recent developments in clinical decision support [1,2]. These models, trained on vast biomedical corpora, have demonstrated remarkable capacity to encode clinical knowledge and generate contextually appropriate medical responses [1]. Their performance on standardized assessments has advanced rapidly, with LLMs approaching or exceeding passing thresholds on medical licensing examinations [2], and chatbot-generated replies to patient questions have, in some settings, been rated comparably to or more favorably than physician replies for quality and empathy [3]. However, their clinical deployment carries substantial limitations, including the generation of inaccurate or fabricated outputs (commonly termed “hallucinations”), variable output quality, and the absence of transparent reasoning pathways [4]. A further concern is that these models can express unwarranted confidence in domains beyond their reliable competence, complicating their unsupervised use in specialized oncologic practice [5]. Safe integration therefore requires that AI complement rather than replace human expertise [6].

Multidisciplinary tumor boards (MDTs) represent the gold standard for complex oncologic decision-making, integrating expertise across multiple disciplines to formulate individualized treatment strategies [7,8]. Against this benchmark, generative AI platforms have demonstrated growing capacity for treatment recommendation generation, mutation interpretation, and clinical trial matching in oncology [9]. However, systematic validation of LLM outputs against real-world MDT decisions remains limited, and the reliability of these models in complex, individualized clinical scenarios has not been adequately established.

Gynecologic oncology presents particular challenges for AI-based decision support owing to the heterogeneous spectrum of malignancies it encompasses—ovarian, endometrial, cervical, and rare tumors—each with distinct molecular profiles, treatment paradigms, and complex considerations such as fertility-sparing approaches and genetic testing for hereditary cancer syndromes. Interest in applying LLMs to this field has grown accordingly, spanning both patient-facing information and guideline-concordant decision support [10]. A recent systematic review concluded that, although LLMs show promise in gynecologic oncology, critical evidence gaps persist regarding complex case-based decision-making and reproducibility [11]. Existing studies have predominantly utilized earlier model versions and limited sample sizes: Meyer et al. evaluated GPT-4 in 60 gynecologic and senologic cases [12], Psilopatis et al. demonstrated feasibility but noted discrepancies in complex cases [13], and Gumilar et al. found variable accuracy across three LLMs on gynecologic cancer vignettes [14]. Most recently, Pergialiotis et al. evaluated ChatGPT-5 against MDT decisions in 599 gynecologic cancer cases, reporting high overall concordance but recurrent discrepancies in advanced and recurrent disease, with staging disagreements predominating [15]. However, that study entered each case only once and therefore did not assess the reproducibility of AI outputs, nor did it quantify performance within precision-oncology domains such as hereditary-cancer genetic testing and fertility-sparing management. The reliability of a current-generation model under repeated querying, and its behavior across these precision-oncology domains, thus remain undefined.

This study aimed to evaluate the concordance between ChatGPT-5 and MDT recommendations in gynecologic oncology, assess reproducibility across repeated independent queries, and identify specific clinical domains of AI–expert divergence, using a blinded, multi-observer design across 242 gynecologic cancer cases. By coupling a quantitative, blinded dual-rater scoring framework with a formal same-day reproducibility assessment and pre-specified subgroup analyses, we sought to move beyond a single global concordance estimate and characterize where, and how reliably, a current-generation LLM aligns with multidisciplinary expert consensus.

## 2. Materials and Methods

### 2.1. Study Design

This retrospective, cross-sectional, comparative study was conducted at the Department of Gynecologic Oncology, Çukurova University Faculty of Medicine, Adana, Turkey. The study evaluated concordance between ChatGPT-5 (OpenAI, San Francisco, CA, USA; training data cutoff: June 2024) and institutional gynecologic oncology MDT decisions. The study period extended from January 2024 to December 2025. The study design was informed by the DECIDE-AI framework for early-stage evaluation of AI-driven decision support systems [16], and reporting followed, where applicable, the REFINE consensus checklist for foundation and large language models in medical research [17].

### 2.2. Patient Population

A total of 242 consecutive gynecologic cancer cases requiring multimodal treatment planning were included: endometrial (*n* = 102, 42.1%), ovarian (*n* = 85, 35.1%), cervical (*n* = 40, 16.5%), and rare tumors (*n* = 15, 6.2%). Cases with incomplete staging or those discussed solely for palliative management were excluded. Cases were categorized as early-stage (FIGO I–II) or advanced-stage (III–IV) for subgroup analysis. Clinical characteristics are summarized in Table 1.

### 2.3. MDT Evaluation and AI Recommendation Generation

The MDT convened weekly, comprising specialists from medical oncology, gynecologic oncology, radiation oncology, radiology, pathology, and nuclear medicine. Consensus recommendations served as the reference standard.

For each case, a standardized clinical summary was constructed by a researcher blinded to the MDT decision, including demographics, ECOG performance status, comorbidities, FIGO stage, histopathology, molecular markers (mismatch repair/MSI status, *BRCA1/2* mutation, HER2, PD-L1 where available), radiological findings, and patient preferences. Summaries were entered into ChatGPT-5 using a structured prompt template (Supplementary Table S1) requesting guideline-concordant recommendations based on current NCCN and ESGO standards. The prompt assigned ChatGPT-5 the role of a gynecologic oncology specialist, instructed it to generate individualized treatment recommendations, and required a structured output comprising a recommended treatment plan, rationale, alternatives, genetic testing indications, and follow-up schedule. Each case was entered as a new conversation with no follow-up prompts. To maximize standardization, identical template wording and field structure were applied verbatim across all cases, and default model settings were used without adjustment of any user-modifiable parameters.

### 2.4. Blinded Evaluation

Both MDT and AI recommendations were independently evaluated by two blinded medical oncologists (Ertugrul Bayram and Berksoy Sahin), who were not involved in the MDT discussions or the AI data entry process, using a 5-point Likert scale (1 = completely inappropriate; 5 = completely appropriate), assessing guideline adherence, clinical appropriateness, treatment plan completeness, and molecular/genetic factor consideration. Raters were blinded to the source (MDT or AI) of each recommendation; both were medical oncologists, a point we revisit in the study limitations. A composite performance score (CPS) was calculated as (mean Likert score/5) × 100.

### 2.5. Reproducibility Assessment

Each case was queried at three separate time points within the same calendar day using independent conversations. Binary consistency (identical core recommendations across all three queries) and a reproducibility score (5-point Likert; 1 = completely different, 5 = identical) were assessed.

### 2.6. Statistical Analysis

Inter-rater reliability and MDT–AI concordance were assessed using Cohen’s kappa (κ) [18], interpreted according to the Landis and Koch classification (0.21–0.40 = fair, 0.41–0.60 = moderate, 0.61–0.80 = substantial, 0.81–1.00 = almost perfect) [19]. CPS comparisons used the Wilcoxon signed-rank test; subgroup comparisons used Mann–Whitney U and chi-squared/Fisher’s exact tests. All analyses were two-sided (*p* < 0.05). Software: SPSS version 25.0 (IBM Corp., Armonk, NY, USA).

### 2.7. Ethical Considerations

Approved by the Çukurova University Faculty of Medicine Non-Interventional Clinical Research Ethics Committee (decision no. 81, meeting no. 164, dated 13 March 2026). Informed consent was waived (retrospective, anonymized design). Conducted per the Declaration of Helsinki.

## 3. Results

### 3.1. Patient Characteristics

Of 242 included cases, the median age was 56 years (range 24–79). By FIGO stage, 145 (59.9%) were early-stage (I–II) and 97 (40.1%) were advanced-stage (III–IV). The MDT recommended genetic testing in 68 cases (28.1%), fertility-sparing treatment was considered in 38 (15.7%), and novel agents or immunotherapy were relevant in 52 (21.5%) (Table 1).

### 3.2. Inter-Rater Reliability and Concordance

Inter-rater reliability was substantial for MDT (κ = 0.761, *p* < 0.001) and almost perfect for AI recommendations (κ = 0.814, *p* < 0.001). Concordance between MDT and ChatGPT-5 was fair (Rater 1: κ = 0.258; Rater 2: κ = 0.334; both *p* < 0.001) (Table 2).

### 3.3. Comparative Performance

**Figure 1:**
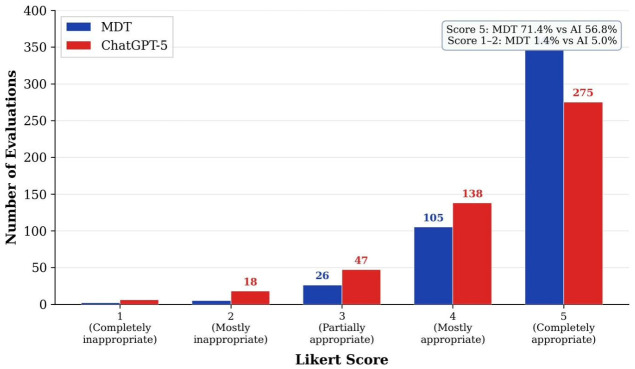
Distribution of Likert scores for MDT and AI-generated recommendations. Pooled across both raters (*n* = 484 evaluations per source: 242 cases × 2 raters). MDT recommendations received a score of 5 (completely appropriate) in 71.4% of evaluations versus 56.8% for AI. Scores of 1–2 were assigned in 1.4% of MDT versus 5.0% of AI evaluations.

CPS were significantly higher for MDT versus AI (Rater 1: 93.8% ± 5.2 vs. 89.4% ± 6.7; Rater 2: 93.4% ± 5.5 vs. 89.7% ± 6.4; both *p* < 0.001). By tumor type, AI achieved the highest CPS for endometrial cancer (91.2% ± 5.8), followed by cervical (89.6% ± 6.3), ovarian (88.4% ± 7.1), and rare tumors (84.2% ± 8.4; Kruskal–Wallis *p* = 0.014). MDT scores were homogeneous across tumor types (*p* = 0.582). MDT recommendations received a Likert score of 5 in 71.4% of pooled evaluations, compared with 56.8% for AI (Table 3, Figure 1).

### 3.4. Reproducibility

**Figure 2:**
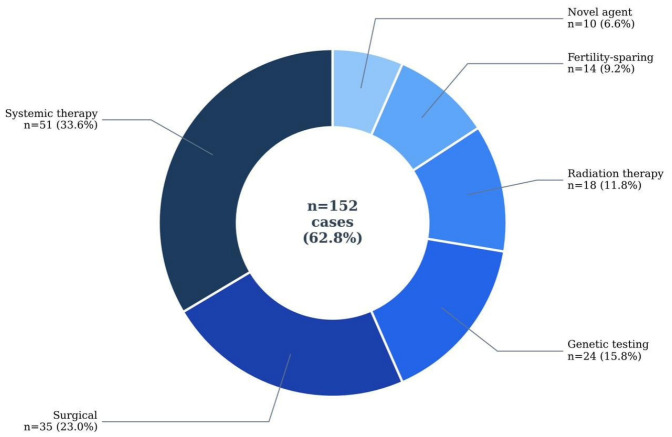
Distribution of inconsistency domains among cases with variable AI outputs (*n* = 152). Among 152 cases (62.8%) exhibiting at least one clinically meaningful variation: systemic therapy selection (33.6%), surgical management (23.0%), genetic testing (15.8%), radiation therapy (11.8%), fertility-sparing (9.2%), novel agents (6.6%).

Full consistency was observed in only 90 of 242 cases (37.2%). The mean reproducibility score was 4.06 ± 0.87. Among 152 inconsistent cases, variations most frequently involved systemic therapy selection (33.6%), surgical management (23.0%), genetic testing recommendations (15.8%), radiation therapy indications (11.8%), fertility-sparing specifics (9.2%), and novel agent integration (6.6%) (Figure 2).

### 3.5. Subgroup Analyses

**Disease stage.** Early-stage cases achieved higher CPS (91.6% ± 5.4 vs. 87.2% ± 7.3; *p* = 0.008) and consistency rates (42.8% vs. 28.9%; *p* = 0.031) compared with advanced-stage cases.

**Genetic testing.** Cases requiring genetic testing (*n* = 68) had lower CPS (86.8% ± 7.8 vs. 90.6% ± 5.9; *p* = 0.006) and reproducibility (26.5% vs. 41.4%; *p* = 0.028). Deficiencies included failure to recommend germline *BRCA1/2* testing, inconsistent Lynch syndrome screening, and omission of somatic HRD testing.

**Fertility-sparing.** Cases involving fertility preservation (*n* = 38) received lower CPS (87.4% ± 7.2 vs. 90.2% ± 6.2; *p* = 0.018).

**Novel therapeutics.** Cases requiring novel agents (*n* = 52) showed the lowest CPS (85.6% ± 8.1 vs. 90.8% ± 5.6; *p* = 0.003) and reduced consistency (25.0% vs. 40.5%; *p* = 0.034) (Table 4).

### 3.6. Patterns of Discordance

In ovarian cancer, predominant discordance involved primary debulking surgery versus neoadjuvant chemotherapy decisions and PARP inhibitor maintenance selection. In endometrial cancer, adjuvant therapy for intermediate-risk disease was the main source of divergence. In cervical cancer, radical surgery versus chemoradiation selection and fertility-sparing appropriateness were key areas of discordance. In rare tumors, AI provided generic algorithms without accounting for specific histological subtypes. Detailed discordance patterns are presented in Supplementary Table S2.

## 4. Discussion

This study evaluated ChatGPT-5 against gynecologic oncology tumor board decisions with, to our knowledge, the first dedicated same-day reproducibility assessment and a blinded, quantitative dual-rater scoring framework applied in this setting. Three principal findings emerged: fair concordance (κ = 0.258–0.334) with a consistent CPS advantage for MDT of approximately 4 points, poor reproducibility (37.2% full consistency), and significantly inferior AI performance in advanced-stage disease, genetic testing, fertility-sparing management, and novel therapeutics—notably in the clinical domains where decision support would arguably be most needed. The fair concordance observed is particularly noteworthy given the superficially similar quality scores between MDT and AI, suggesting that aggregate performance metrics may mask clinically meaningful divergence in individual case recommendations. Furthermore, in the fertility-sparing subgroup, AI frequently provided overly conservative recommendations or failed to recognize contraindications, underscoring the challenges current LLMs face in navigating nuanced, patient-centered decision-making.

Our concordance rates are consistent with findings from studies in similarly complex clinical settings. In breast cancer, Liao et al. reported that only 46% of ChatGPT-4.0 outputs fully aligned with expert recommendations across 362 patients, with lower performance in advanced-stage disease and molecular marker-dependent scenarios [20]. In contrast, Büyükceran et al. achieved 93.9% concordance in 33 patients using highly structured inputs [21], highlighting the substantial impact of prompt design and case complexity on measured agreement. In urologic oncology, De la Torre-Trillo et al. reported a kappa of 0.61 [22], and in sarcoma, Dehdab et al. found deficiencies in treatment sequencing and chemotherapy selection despite reasonable overall performance [23]. In mixed solid tumors, Dogan et al. observed 76.4% agreement, with most discrepancies arising in cases requiring individualized judgment [24]. Notably, Karabuğa et al., using a similar methodological design with two blinded raters and a 5-point Likert scale, reported concordance values of κ = 0.211–0.376 between ChatGPT-4o and an in-house tumor board across 102 patients with diverse cancer types [25]—values strikingly similar to those observed in our study (κ = 0.258–0.334) despite the use of a newer-generation model and a tumor type-specific cohort. Our findings align closely with the most directly comparable study: Pergialiotis et al. evaluated the same model (ChatGPT-5) against MDT decisions in a larger cohort of 599 cases and likewise found that concordance deteriorated in advanced and recurrent disease, with staging and multimodal decisions driving discordance [15]. That concurrent, same-model study entered each case only once; our reproducibility analysis adds the dimension it did not address, demonstrating that even a current-generation model yields inconsistent recommendations upon repeated querying. Moreover, the convergence of our ChatGPT-5 results with the earlier GPT-4o data of Karabuğa et al. indicates that advancing model generation does not, by itself, substantially narrow the LLM–expert gap. Comparative work across models reinforces this interpretation: contextualization and model selection materially affect oncologic output quality [26], and structured chatbot evaluations in other complex domains, such as thyroid nodule and papillary thyroid cancer management, report a similar pattern of promise tempered by inconsistency in nuanced cases [27]. In head and neck cancer, Hack et al. demonstrated that retrieval-augmented generation (RAG) approaches yielded superior guideline-concordant recommendations compared with standard LLM outputs [28]. Within gynecologic oncology specifically, the systematic review by Rosati et al. reported concordance rates of 70–75% for ChatGPT-4 with international guidelines, with declining performance in nuanced clinical scenarios [11]. These findings collectively confirm an inverse relationship between clinical complexity and AI concordance.

To make the relationship between our study and the most directly comparable, concurrent same-model work explicit, Table 5 summarizes how the present study extends Pergialiotis et al. [15]—addressing the reproducibility and precision-oncology questions their single-query design left open while independently corroborating their central finding.

The reproducibility findings are among the most clinically consequential observations of this study. ChatGPT-5 produced fully consistent recommendations in only 37.2% of cases upon three same-day queries, closely paralleling the finding of 32% consistency reported by Liao et al. in breast cancer [20]. Notably, reproducibility was significantly lower in cases involving genetic testing in both studies (ours: *p* = 0.028; Liao: *p* = 0.013), suggesting that genomic aspects of decision-making represent a particularly vulnerable domain for current LLMs. Kaplan et al., evaluating ChatGPT Omni across 700 gynecologic oncology questions at three-month intervals, found that while temporal stability was modest, consistency remained suboptimal for complex clinical scenarios [29]. An advisory tool that generates meaningfully different recommendations upon consecutive queries of identical scenarios is fundamentally incompatible with the principles of consistent, evidence-based decision-making.

Advanced-stage disease was associated with significantly lower CPS and consistency, consistent with findings across multiple tumor types. In pancreatic oncology, Mergen et al. demonstrated that despite 78.6% overall accuracy in tumor board decisions, an LLM failed to identify any neoadjuvant chemotherapy candidates (recall = 0.00), illustrating critical safety limitations that may be masked by favorable aggregate metrics [30]. Regarding genetic testing, the complexity of current ASCO guidelines for germline and somatic testing in epithelial ovarian cancer [31] and the rapidly evolving landscape of biomarker-driven therapy likely exceed what training data alone can capture. Griewing et al. consistently identified genetic testing as the domain of lowest LLM–MDT concordance across five model versions in breast cancer [32], a finding that our data corroborate.

The poor performance in fertility-sparing and novel therapeutics domains reflects that these areas are characterized by rapidly evolving evidence and individualized risk–benefit calculations that extend beyond algorithmic guideline application. Current consensus guidelines for ovarian [33], endometrial [34], and cervical cancer [35] increasingly incorporate molecular classification, biomarker-driven therapy, and fertility-sparing algorithms requiring contextualized interpretation—capabilities that remain underdeveloped in current LLMs. Furthermore, the training data cutoff of ChatGPT-5 (June 2024) may have limited its ability to incorporate the most recent guideline updates and emerging therapeutic agents approved after this date, particularly in the rapidly evolving landscape of molecular-targeted and immunotherapy-based regimens in gynecologic oncology. These domain-specific deficiencies suggest concrete avenues for refinement rather than wholesale rejection of LLM support. Retrieval-augmented generation grounded in current NCCN and ESGO guideline corpora, domain-specific fine-tuning on curated gynecologic oncology cases, and deployment within structured clinical-decision-support platforms have each been associated with improved guideline concordance in oncology settings [28,36], and represent plausible strategies to address the precision-oncology gaps identified here.

Translating these findings into practice requires careful attention to clinical-workflow integration. In its current form, ChatGPT-5 appears better suited to a preparatory, human-in-the-loop role—structuring case summaries, generating checklists of guideline-relevant considerations, or flagging genetic-testing and clinical-trial opportunities ahead of the MDT—than to autonomous recommendation. Principal barriers to safe deployment include the limited reproducibility demonstrated here, unresolved questions of clinical accountability, data-governance and privacy requirements, and the difficulty of keeping model knowledge current with rapidly evolving guidelines. Realizing benefit will therefore depend not only on model performance but also on governance frameworks, clinician training, and workflow redesign that preserve expert oversight while capturing the efficiency gains AI may offer [9,36].

This study has limitations. As a single-institution, retrospective analysis, our concordance estimates reflect the practice patterns of one academic MDT and may not generalize to other centers or case-mixes; they should be interpreted as institution-specific. The two independent raters, although blinded, were both medical oncologists, which imposes a specialty-specific evaluative lens; high inter-rater reliability (κ = 0.761–0.814) mitigates but does not eliminate this concern. Additional limitations include the single-LLM design, same-day-only reproducibility assessment, unstandardized prompt variation, limited statistical power for the rare tumor subgroup (*n* = 15), and the use of concordance as a surrogate without patient outcome data. The composite performance score used in this study is a pragmatic, non-validated metric designed for this analysis; however, its derivation from widely used Likert scales and its transparent formula enhance interpretability. Nevertheless, this study provides a blinded, multi-observer comparison and, to our knowledge, the first to combine systematic same-day reproducibility assessment with quantitative blinded dual-rater scoring across all major gynecologic tumor types, including rare entities; the design was informed by the DECIDE-AI framework [16]. Future research should prioritize prospective, multi-center evaluations across diverse healthcare settings, incorporate multidisciplinary raters (including surgeons and radiologists) with reporting of inter-rater variability across specialties, multi-model comparative evaluations, explore RAG and fine-tuning approaches—which demonstrated superiority in head and neck oncology [28] and hold promise for addressing the limitations identified herein [9]—assess reproducibility over extended time intervals (weeks to months), and evaluate whether AI-augmented MDT workflows translate into measurable improvements in patient outcomes. Until then, human expertise remains indispensable, and AI should be regarded as a complement to—not a substitute for—the multidisciplinary tumor board.

## 5. Conclusions

In this blinded, multi-observer study of 242 gynecologic cancer cases, ChatGPT-5 demonstrated fair concordance with MDT recommendations (κ = 0.258–0.334) and full reproducibility in only 37.2% of cases. Performance was significantly inferior in advanced-stage disease, genetic testing, fertility-sparing management, and novel therapeutics integration. Notably, a concurrent study evaluating the same model in a larger cohort reached a convergent conclusion, indicating that architectural advancement alone does not bridge the gap between LLM outputs and expert consensus. At present, ChatGPT-5 may serve as an educational or preparatory adjunct, but human expertise remains indispensable for the individualized management of complex gynecologic malignancies.

## Figures and Tables

**Table 1:** Clinical and pathological characteristics of the study population (*n* = 242).

Characteristic	Total (*n* = 242)	Endometrial (*n* = 102)	Ovarian (*n* = 85)	Cervical (*n* = 40)	Rare (*n* = 15)
Age, median (range), y	56 (24–79)	61 (32–79)	54 (28–76)	46 (24–72)	52 (31–74)
**ECOG Performance Status**					
0	98 (40.5)	42 (41.2)	30 (35.3)	20 (50.0)	6 (40.0)
1	112 (46.3)	48 (47.1)	40 (47.1)	16 (40.0)	8 (53.3)
≥2	32 (13.2)	12 (11.8)	15 (17.6)	4 (10.0)	1 (6.7)
**FIGO Stage**					
I	106 (43.8)	65 (63.7)	15 (17.6)	18 (45.0)	8 (53.3)
II	39 (16.1)	12 (11.8)	8 (9.4)	14 (35.0)	5 (33.3)
III	67 (27.7)	18 (17.6)	42 (49.4)	6 (15.0)	1 (6.7)
IV	30 (12.4)	7 (6.9)	20 (23.5)	2 (5.0)	1 (6.7)
Early (I–II)	145 (59.9)	77 (75.5)	23 (27.1)	32 (80.0)	13 (86.7)
Advanced (III–IV)	97 (40.1)	25 (24.5)	62 (72.9)	8 (20.0)	2 (13.3)
**Grade**					
1 (well diff.)	55 (22.7)	32 (31.4)	8 (9.4)	10 (25.0)	5 (33.3)
2 (moderately diff.)	95 (39.3)	42 (41.2)	25 (29.4)	20 (50.0)	8 (53.3)
3 (poorly diff.)	82 (33.9)	24 (23.5)	48 (56.5)	8 (20.0)	2 (13.3)
Not graded	10 (4.1)	4 (3.9)	4 (4.7)	2 (5.0)	0 (0.0)
**Molecular Markers Tested**					
MMR/MSI assessed	85 (35.1)	62 (60.8)	18 (21.2)	3 (7.5)	2 (13.3)
dMMR/MSI-H	12 (14.1 †)	10 (16.1 †)	2 (11.1 †)	0	0
*BRCA1/2* tested	62 (25.6)	8 (7.8)	48 (56.5)	2 (5.0)	4 (26.7)
Pathogenic variant	15 (24.2 †)	1 (12.5 †)	12 (25.0 †)	0	2 (50.0 †)
**Clinical Considerations**					
Genetic testing rec.	68 (28.1)	18 (17.6)	38 (44.7)	6 (15.0)	6 (40.0)
Fertility-sparing	38 (15.7)	14 (13.7)	8 (9.4)	12 (30.0)	4 (26.7)
Novel agents/IO	52 (21.5)	16 (15.7)	26 (30.6)	6 (15.0)	4 (26.7)
**Comorbidities**					
None	88 (36.4)	30 (29.4)	32 (37.6)	20 (50.0)	6 (40.0)
1 comorbidity	82 (33.9)	36 (35.3)	30 (35.3)	10 (25.0)	6 (40.0)
≥2 comorbidities	72 (29.8)	36 (35.3)	23 (27.1)	10 (25.0)	3 (20.0)

Values are *n* (%) unless otherwise stated. † Percentage of those tested. dMMR = deficient mismatch repair; MSI-H = microsatellite instability-high; ECOG = Eastern Cooperative Oncology Group; FIGO = International Federation of Gynecology and Obstetrics; diff. = differentiated; IO = immunotherapy; rec. = recommended.

**Table 2:** Inter-rater reliability and concordance between MDT and ChatGPT-5 recommendations.

Comparison	κ	*p*-Value	Interpretation
**Inter-rater reliability (Rater 1 vs. Rater 2)**			
MDT recommendations	0.761	<0.001	Substantial
AI recommendations	0.814	<0.001	Almost perfect
**MDT–AI concordance**			
Rater 1	0.258	<0.001	Fair
Rater 2	0.334	<0.001	Fair

κ = Cohen’s kappa. Interpretation according to Landis and Koch [19]: 0.21–0.40 = fair; 0.61–0.80 = substantial; 0.81–1.00 = almost perfect. MDT = multidisciplinary tumor board; AI = artificial intelligence.

**Table 3:** Composite performance scores: MDT versus ChatGPT-5, overall and by tumor type.

Category	MDT CPS (%)	AI CPS (%)	Δ (Points)	*p*-Value
**Overall (by rater)**				
Rater 1	93.8 ± 5.2	89.4 ± 6.7	4.4	<0.001
Rater 2	93.4 ± 5.5	89.7 ± 6.4	3.7	<0.001
**By tumor type (pooled raters)**				
Endometrial (*n* = 102)	94.1 ± 4.8	91.2 ± 5.8	2.9	<0.001
Ovarian (*n* = 85)	93.2 ± 5.6	88.4 ± 7.1	4.8	<0.001
Cervical (*n* = 40)	93.8 ± 5.1	89.6 ± 6.3	4.2	<0.001
Rare (*n* = 15)	92.4 ± 6.2	84.2 ± 8.4	8.2	0.002
*Kruskal–Wallis*	*p = 0.582*	*p = 0.014*	—	—

CPS = composite performance score = (mean Likert score/5) × 100. Values are mean ± SD. Δ = mean difference (MDT − AI). Wilcoxon signed-rank test for paired comparisons; Kruskal–Wallis test for across-group comparisons.

**Table 4:** Subgroup analysis of AI performance by clinical variables.

Variable	*n*	AI CPS (%)	*p* *	Full Consistency *n*/*N* (%)	*p* †
**Disease Stage**					
Early (I–II)	145	91.6 ± 5.4	0.008	62/145 (42.8)	0.031
Advanced (III–IV)	97	87.2 ± 7.3		28/97 (28.9)	
**Genetic Testing Recommended**					
Yes	68	86.8 ± 7.8	0.006	18/68 (26.5)	0.028
No	174	90.6 ± 5.9		72/174 (41.4)	
**Fertility-Sparing Considered**					
Yes	38	87.4 ± 7.2	0.018	10/38 (26.3)	0.122
No	204	90.2 ± 6.2		80/204 (39.2)	
**Novel Therapeutics Required**					
Yes	52	85.6 ± 8.1	0.003	13/52 (25.0)	0.034
No	190	90.8 ± 5.6		77/190 (40.5)	

CPS values are mean ± SD. Full consistency = identical core treatment recommendations across all three queries. * Mann–Whitney U test. † χ^2^ test or Fisher’s exact test.

**Table 5:** Comparison of the present study with the concurrent same-model study of Pergialiotis et al.

Dimension	Pergialiotis et al., Cancers 2026 [15] (Concurrent Same-Model Study)	Present Study
Model evaluated	ChatGPT-5 (599 cases, single center)	ChatGPT-5 (242 cases, independent institution)—independent corroboration
Reproducibility	Each case entered once; not assessed	Same-day test–retest (3 independent queries/case); full consistency in only 37.2% of cases
Evaluation method	Binary major/minor discrepancy classification (gynecologic oncologist + data scientist)	Blinded two-clinician 5-point Likert appropriateness scoring + CPS; both MDT and AI arms scored
Precision-oncology domains	Staging, surgery, systemic and targeted therapy	Pre-specified subgroups incl. hereditary genetic testing (*p* = 0.006), fertility-sparing (*p* = 0.018), novel therapeutics (*p* = 0.003)—all significant
Tumor coverage	Cervical, endometrial, ovarian, vulvar	Endometrial, ovarian, cervical, plus rare tumors
Complementary message	High overall concordance; discrepancies in advanced/recurrent disease	Independently corroborates complexity-dependent decline and adds that outputs are not reproducible, reinforcing that model generation alone is insufficient for autonomous use

LLM, large language model; MDT, multidisciplinary tumor board; CPS, composite performance score; κ, Cohen’s kappa.

## Data Availability

Data are available from the corresponding author upon reasonable request.

## Supplementary Materials

The following supporting information can be downloaded at: https://www.mdpi.com/article/10.3390/jcm15124451/s1, Supplementary Table S1: Structured prompt template used for ChatGPT-5 case input; Supplementary Table S2: Detailed patterns of MDT–AI discordance by tumor type.
