# Supplementary material for: Feasibility and Concordance of a Large Language Model (ChatGPT-5) as a Clinical Decision Support Tool in Gynecologic Oncology Tumor Boards: A Blinded, Multi-Observer Study
Source: J Clin Med. 2026 Jun 9;15(12):4451. doi: 10.3390/jcm15124451 (PMC13301218; doi:10.3390/jcm15124451)
Supplement: Supplementary file 1 [file jcm-15-04451-s001.zip › jcm-4291998-supplementary.pdf]

Supplementary Materials

Feasibility and Concordance of a Large Language Model (ChatGPT-5) as a Clinical Decision Support Tool in Gynecologic Oncology Tumor Boards: A Blinded, Multi-Observer Study

Supplementary Table S1. Structured prompt template used for ChatGPT-5 case input.

SYSTEM PROMPT:

"You are a gynecologic oncology specialist. Based on the following clinical information, provide a comprehensive, individualized treatment recommendation in accordance with current NCCN and ESGO guidelines. Include recommendations for surgery, systemic therapy, radiation therapy, genetic testing/counseling, fertility considerations (if applicable), and follow-up. Provide your rationale for each recommendation."

STRUCTURED CLINICAL INPUT:

Field	Content
**Patient Demographics:**	[Age] / [Sex] / [BMI]
**ECOG Performance Status:**	[0–4]
**Relevant Comorbidities:**	[List or 'None']
**Primary Diagnosis:**	[Tumor type] / [Histological subtype]
**FIGO Stage:**	[Stage with substage]
**Grade:**	[1/2/3 or High-grade/Low-grade]
**Pathology Details:**	[Tumor size, LVSI, depth of invasion, margin status, LN status]
**Molecular/IHC Profile:**	[ER/PR, HER2, MMR/MSI, BRCA1/2, PD-L1, HRD — as available]
**Radiological Findings:**	[Key imaging findings]
**Prior Treatments:**	[Surgery/chemo/RT — if any]
**Patient Preferences:**	[Fertility preservation, treatment preferences]
**Specific Clinical Question:**	[MDT discussion question, if any]

OUTPUT INSTRUCTIONS:

"Provide your recommendation in the following format: (1) Recommended treatment plan, (2) Rationale for each component, (3) Alternative options if applicable, (4) Recommended genetic testing/counseling if indicated, (5) Follow-up schedule."

Each case was entered as a new, independent conversation. No follow-up prompts, clarifications, or guidance were provided beyond the initial structured input.

Supplementary Table S2. Detailed patterns of MDT–AI discordance by tumor type.

Tumor Type	Discordance Domain	Specific Pattern	Clinical Implication
**Ovarian**	Primary surgery vs. NACT	AI defaulted to NACT in borderline-resectable cases where MDT recommended PDS	Potential delay of optimal cytoreduction
	PARP inhibitor maintenance	Inconsistent selection/duration; variable BRCA/HRD integration	Suboptimal maintenance strategy
	Genetic testing	Failure to recommend germline BRCA testing; omission of somatic HRD in HGSC	Missed therapeutic opportunities
**Endometrial**	Adjuvant therapy (intermediate-risk)	Formulaic approach; less responsive to LVSI extent and molecular classification	Over/under-treatment risk
	Molecular classification	Inconsistent application of TCGA/ProMisE subtypes	Failure to individualize risk
	Fertility-sparing	Overly conservative or missed contraindications (high-grade, deep invasion)	Inappropriate candidate selection
**Cervical**	Surgery vs. chemoradiation	Inconsistent recommendations for IB2–IIA (radical surgery vs. definitive CRT)	Wrong modality selection
	Fertility-sparing trachelectomy	Failure to recognize eligibility criteria or contraindications	Missed fertility preservation
**Rare tumors**	Treatment algorithm	Generic management without histology-specific adaptation (vulvar, vaginal, LMS vs. ESS)	Inappropriate generalization
	Evidence integration	Overconfident recommendations despite limited evidence base	Misleading clinical guidance

NACT = neoadjuvant chemotherapy; PDS = primary debulking surgery; HGSC = high-grade serous carcinoma; HRD = homologous recombination deficiency; LVSI = lymphovascular space invasion; TCGA = The Cancer Genome Atlas; CRT = chemoradiation; LMS = leiomyosarcoma; ESS = endometrial stromal sarcoma.
